# Dietary Diversity and Associated Factors Among Pregnant Women in Addis Ababa, Ethiopia, 2021

**DOI:** 10.3389/ijph.2022.1605377

**Published:** 2022-11-03

**Authors:** Aynshet Nega Kebede, Addisu Tadesse Sahile, Bethlehem Chala Kelile

**Affiliations:** ^1^ Department of Outpatient, Felege Meles Health Center, Addis Ababa, Ethiopia; ^2^ Department of Public Health, Unity University, Addis Ababa, Ethiopia

**Keywords:** Ethiopia, women, associated factors, dietary practice, Addis Ababa

## Abstract

**Objective:** This study aimed to assess the dietary diversity and its associated factors among pregnant women in Addis Ababa, Ethiopia, 2021.

**Methods:** An institution-based Cross-sectional was conducted among 320 participants from four health facilities in Addis Ababa selected based on a simple random sampling method from 01 September to 30 December 2021. An Interviewer-administered structured questionnaire was used, following informed consent. Binary (Bivariate and multivariate) logistics regression was applied for the identification factors associated with dietary diversity with their respective 95% confidence interval and less than 5% *p*-value.

**Results:** The prevalence of inadequate dietary diversity during pregnancy was 51.6% (95% CI: 46.1%–57.0%). Being illiterate (AOR: 0.591; 95% CI: 1.88–1.901; *p < 0.05*) and primary education (AOR: 0.347; 95% CI: 0.166–0.728; *p < 0.05*), having poor knowledge (AOR: 0.437; 95% CI: 0.252–0.757; *p* < 0.05) and lower monthly income (AOR: 0.395; 95% CI: 0.184–0.845; *p < 0.05*) were factors associated with inadequate dietary diversity.

**Conclusion:** A higher level of inadequate dietary diversity was reported. Being illiterate, having primary education, having poor knowledge, and having lower monthly income were associated with inadequate dietary diversity during pregnancy. Concerned bodies were suggested to work on the identified factors.

## Introduction

A healthy diet is essential throughout one’s life, but especially during pregnancy. Pregnant women are nutritionally vulnerable due to their increased nutrient demand. Consumption of a variety of dietary sources is important for ensuring optimal maternal and child nutrition, as it prevents nutritional deficiencies and negative consequences [1, 2].

Dietary diversity is about taking varieties of food item and reflected as a proxy measure of micronutrient adequacy [1, 3]. Evidence suggests that dietary diversity promotes nutrient adequacy in women and lowers the risk of a negative birth outcome [4–7]. Dietary diversification is an appealing approach to addressing nutrient deficiency [1].

Micronutrient deficiency remains a major public health concern in low to middle-income countries commonly in reproductive-age women. [1–5], Thus pregnancy augments an additional burden of nutritional requirements for the women and fetus [6–8].

Malnutrition during pregnancy has a long-lasting effect on the physiological development of the fetus through increasing the risk of low birth weight, maternal morbidity and mortality, preterm delivery, and intrauterine growth retardation [9–12].

Optimal nutrient intake during pregnancy reduces the risk of preterm baby, low birth weight, infant mortality, and small for gestational age [13–15]. Moreover, in women who are underweight during their pregnancy supplementation of multiple micronutrients before 20 weeks of gestation reduces the risk of a preterm baby [16–18].

Understanding the enormous benefits of a healthy diet and adequate micronutrient supplementation during pregnancy [19, 20], dietary diversity is one of the best-recommended strategies for the improvement of dietary adequacy and increased food groups in their daily consumption [21]. Thus, dietary diversity refers to the number of different groups of food consumed over some time [22, 23].

Though evidence suggests that the prevalence of nutritional deficiency during pregnancy was higher in Africa [24–26], including Ethiopia, dietary diversification is an essential tool to reduce nutritional deficiencies [27].

To the best of the researcher’s knowledge, the level of dietary diversity and its associated factors during pregnancy was not well investigated in the study settings. Therefore; this study would hopefully fill the existing gap in the literature.

## Methods

### Participants and Study Design

The institutional-based cross-sectional study design was conducted among 320 participants from the selected health facilities at Addis Ketema Sub-City of Addis Ababa from 01 September to 30 December 2021. The study received Ethical approval from Santé Medical College; a research review ethics committee and applied to the respective Health Facilities. All the participants were provided written informed consent.

**FIGURE 1:**
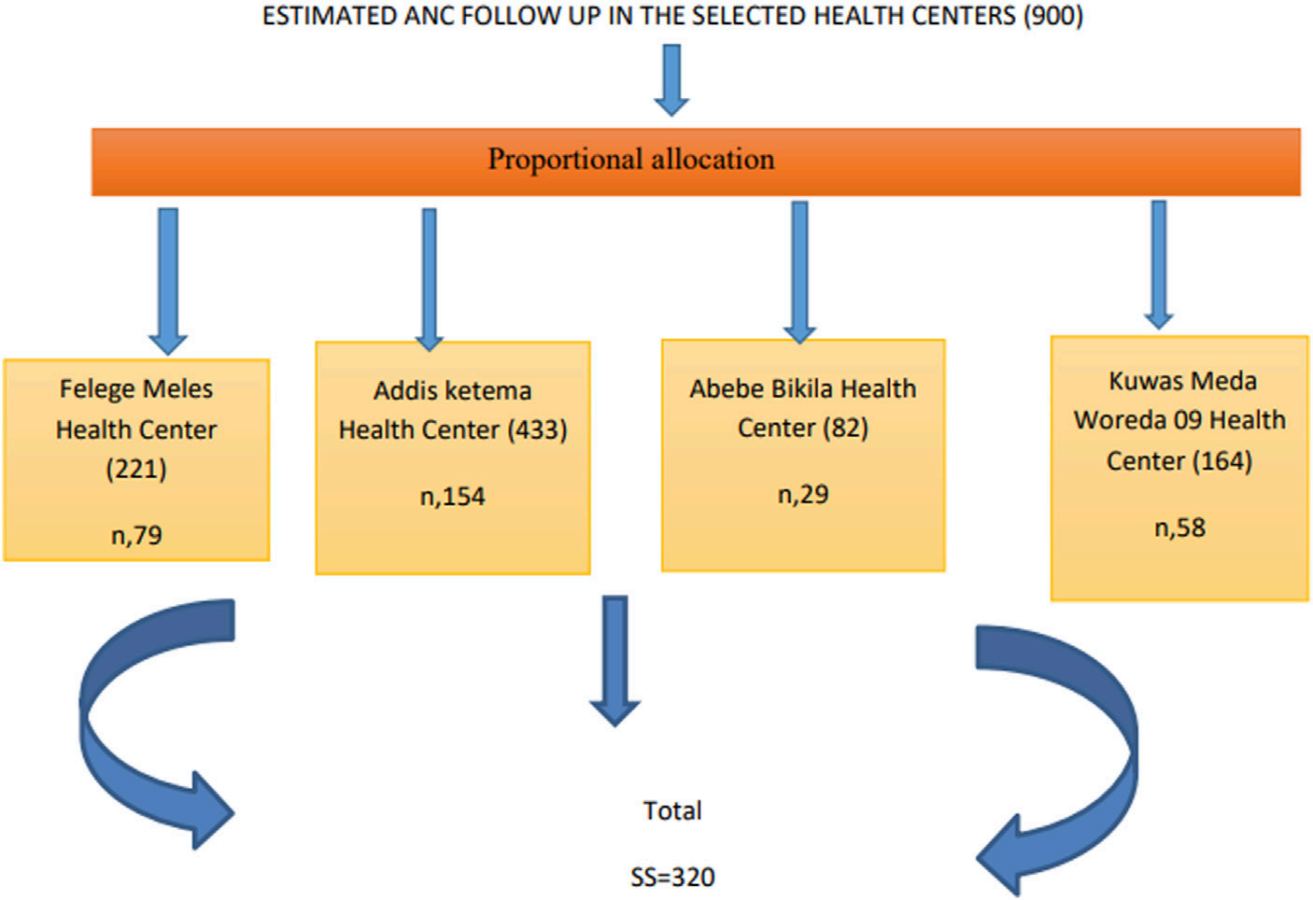
Sampling Procedure of the study participants (Addis Ababa, Ethiopia, 2021).

The source population was all pregnant women in Addis Ketema Sub-City, whereas the study population was pregnant women having had ANC follow-up at the selected health facilities; namely; Addis Ketema, Abebe Bikila, Kuwas Meda, and Felege Meles health centers, during the study period. Pregnant women who were available, willing, and free from any severe medical conditions were included in the study. The researcher selected the health facilities by simple random sampling method, after list of all health facilities was determined from the sub city likewise simple random sampling techniques was employed for the selection of the study participants ones their lists were identified from their medical records at respective facilities. Proportional allocation was made based on the health centers monthly follow-up (Figure 1).

The sample size was determined based on a single population proportion, with the premise that the magnitude of dietary diversity from Shashemene was 25.4% [28], 95% confidence interval of 1.96, and a 5% margin of error. The final sample was 320 inclusive of a 10% non-response rate.

Data were gathered with a pre-tested interviewer-administered questionnaire. The questionnaire was developed by reviewing related pieces of literature [29–32] and then given for two senior researchers working in academic institutions and their inputs were incorporated into the final tool.

### Definitions of Concepts

The outcome variable (Dietary diversity) was measured as adquate and inadequate. A minimum dietary diversity score (MDD-W) was used to calculate the sum of the number of different food groups consumed by the pregnant women in the 24 Hours before the assessment. The MDD-W indicator was based on the 10-food group’s women’s dietary diversity score (MDDW-10). These food groups are starch staples (grains, white roots and tubers, and plantains); vitamin A-rich vegetables and fruits; dark green leafy vegetables; other vegetables; other fruits; flesh foods (meat, fish, poultry, and liver/organ meats); eggs; pulses/legumes; nuts and seeds; and dairy products. MDD-W is based on a 24-h dietary recall period [33]. The women were asked to recall all foods consumed from the above food groups on the previous day. Responses were recorded as “yes” or “no.”A “yes” response was scored as “1,” and a “no” response was scored as ‘0’. The scores summed up to yield the women’s MDD-W score. The dietary diversity scores were then classified as inadequate and adequate dietary diversity based on the MDD-W. Women having a diversity score of less than 5 were classified as having low dietary diversity and scores of 5–10 are classified as the high/good dietary diversity scores [34]. Knowledge was measured as true and false questions and categorized as poor versus good knowledge based on median score after constructs forming knowledge were once computed.

### Statistical Analysis

Descriptive statistics were used for the summarization of data. For the identification factors associated with dietary diversity, binary (Bi-variable and multivariable) logistics regression was used, with their respective 95% Confidence Interval (CI) and *p*-value of less than 0.05 as a statistically significant level.

## Results

### Socio Demographic Characteristics

A total of 320 pregnant women participated in this study making a response rate of 100%. The mean age of study participants was 26.98 with a standard deviation of 4.468. The majority (95%) of the participants were married and more than one-third (37.8%) of their husbands attended primary school. Regarding occupation, most (63.1%) of the study participants were housewives. Almost all (94.4%) of the participants got support from their husband (Table 1).

**TABLE 1:** Socio-demographic characteristics of study participants (Addis Ababa, Ethiopia, 2021).

Variables	Categories	Frequency	%
Age in years	<24	88	27.5
	24–34	208	65.0
	≥35	24	7.5
Marital status	Married	306	95.6
	Single/divorced/widowed	14	4.3
Educational status	Illiterate	22	6.9
	Reading and writing	11	3.4
	Primary	121	37.8
	Secondary	104	32.5
	Collage and above	62	19.4
Occupation	Housewife	202	63.1
	Private employment	46	14.4
	Government employment	34	10.6
	Merchant	24	7.5
	Student	5	1.6
	Daily laborer	7	2.2
	Other	2	0.6
Partner level of education	Illiterate	7	2.2
	Reading and writing	9	2.8
	Primary	86	26.9
	Secondary	143	44.7
	Collage and above	75	23.4
Partner occupation	Private employment	99	30.9
	Government employment	47	14.7
	Merchant	92	28.8
	Student	3	0.9
	Daly laborer	60	18.8
	Other	19	5.9
Monthly income	<2,000	49	15.3
	2,000–3,500	70	21.9
	>3,500	201	62.8
Family size	<4	287	89.7
	≥4	33	10.3
Husband support	No	18	5.6
	Yes	302	94.4

### Maternal Characteristics

Most (59.1%) and (58.4%) were multigravidas and started ANC in their first trimester of pregnancy respectively. About three-fourths (77.8%) of the study participants had information about the importance of dietary diversity during pregnancy whereas less than half (42.8%) of respondents had avoided some kind of food during pregnancy. Most (80.3%) of respondents had a meal frequency of three and above per day (Table 2).

**TABLE 2:** Maternal characteristics of the study participants (Addis Ababa, Ethiopia, 2021).

Variables	Category	Frequency	Percentage
Pregnancy	Prim Gravida	131	40.9
	Multigravida	189	59.1
Time of ANC started	First trimester	187	58.4
	Second trimester	115	35.9
	Third trimester	18	5.6
No of ANC visit	1st visit	108	33.8
	2nd visit	98	30.6
	3rd visit	77	24.1
	4th and above visit	37	11.6
Had an information about food diversity		247	77.8
Avoid any food during current pregnancy		137	42.8
Meal frequency per day	≥3 times	257	80.3
	2 times	50	15.6
	<2 times	13	4.1

### Pregnant Women Knowledge of Dietary Diversity

The proportion of women having had a good dietary diversity knowledge during pregnancy was 69.1% (95% CI: 63.7%–74.1%). A large majority of pregnant women (94.1%) knew the importance of food for the growth and development of the fetus during pregnancy, and (93.4%) perceived food is important for fighting infection. More than three fourth (78.8%) of the study participants reported that inadequate diet can cause miscarriage and stillbirth (Table 3).

**TABLE 3:** Dietary diversity knowledge of the study participants (Addis Ababa, Ethiopia, 2021).

Variables	Option	Number	%
Food is important for growth and development of fetus	Yes	301	94.1
Food provides heat, and energy during pregnancy	Yes	294	91.9
Food is important for fighting infection or disease	Yes	299	93.4
Balanced diet is important during pregnancy	Yes	295	92.2
Inadequate diet can cause miscarriage and still Birth	Yes	252	78.8
Carbohydrate source of foods are recommended during pregnancy	Yes	258	80.6
Protein source of foods are recommended during pregnancy	Yes	274	85
Iron source of foods are recommended during pregnancy	Yes	227	70.9
Vitamin A source of foods are recommended during pregnancy	Yes	277	86.6
Iodine source of foods are recommended during pregnancy	Yes	210	65.6

### Prevalence of Inadequate Dietary Diversity

The prevalence of inadequate dietary diversity during pregnancy was 51.6% (95% CI: 46.1%–57.0%). Most (90%) of the study participants reported that they consumed cereal-based crops (maize, sorghum, millet, wheat, barley, and teff) before the survey. Dark green leafy vegetables (kale, swiss chard, and green pepper) and legumes were consumed by 59.7% and 55.3% of the subjects respectively 1 day before the survey. More than half (52.8%) of the study participants reported that they consumed other vitamins A-rich vegetables (pumpkin, carrot, and orange-fleshed sweet potato). Among animal products, milk and milk products were consumed by (50.9%) of the study group whereas meat, poultry, fish, and egg were consumed by 31.9% and 29.4%, respectively (Table 4).

**TABLE 4:** A 24 h food diversity of the study participants (Addis Ababa, Ethiopia, 2021).

Food groups	Frequency	Percentage
Cereals	288	90
Legumes	177	55.3
Nuts and seeds	78	22.4
Milk and Dairy products	163	50.9
Meat, poultry and fish	102	31.9
Egg	94	29.4
Dark Green leafy vegetables	191	59.7
Other vitamin A rich fruits and vegetables	169	52.8
Other vegetables	294	91.9
Other fruits	206	64.4

### Factors Associated With Dietary Diversity Practice

The bivariate logistics regression identified the age of the mother, educational status, knowledge, attitude, and monthly incomes to be the candidate variables for the multivariate logistics regression at a *p*-value of <0.25. The multivariable logistics regression identified maternal educational status, knowledge and monthly income were factors statistically associated with dietary diversity of the mother.

The odds of having had inadequate dietary diversity was higher by 40.9% among illiterates compared to those of college and above (AOR: 0.591; 95% CI: 1.88–1.901; *p* < 0.05). The odds of having inadequate dietary diversity was higher by 65.3% among mothers who had completed primary schooling compared to those of college and above (AOR: 0.347; 95% CI: 0.166–0.728; *p* < 0.05).

The odds of having inadequate dietary diversity was higher by 56.3% among mothers who had poor knowledge compared to good knowledge (AOR: 0.437; 95% CI: 0.252–0.757; *p* < 0.05). The odds of having inadequate dietary diversity was higher by 65.5% among mothers who had monthly income <2000 compared to >3500monthly income (AOR: 0.395; 95% CI: 0.184–0.845; *p* < 0.05) (Table 5).

**TABLE 5:** Factors affecting dietary diversity of the study participants (Addis Ababa, Ethiopia, 2021).

Characteristics	Category	Dietary inadequate	Diversity adequate	COR (95%CI)	AOR (95%CI)
Age in years	<24	55	33	0.840 (0.335–2.1.6)	1.151 (0.369–3.597)
	24–34	96	112	1.633 (0.694–3.845)	1.494 (0.548–4.074)
	≥35	14	10	1	1
Mother education	Illiterate	13	9	0.306 (0.112–0.837)*	0.599 (1.88–1.901)*
	Reading and writing	8	3	0.166 (0.40–0.6941)*	0.337 (0.72–1.581)
	Primary	78	43	0.244 (0.126–0.469)*	0.347 (0.166–0.728)*
	Secondary	47	57	0.536 (0.276–1.041)	0.535 (0.263–1.088)
	College and above	19	43	1	1
Husband support	No	12	6	0.513 (0.188–1.403)	1.57 (0.373–3.588)
	Yes	153	149	1	1
Knowledge	Poor Knowledge	67	32	0.381 (0.231–0.626)*	0.437 (0.252–0.757)*
	Good Knowledge	98	123	1	1
Attitude	Unfavorable Attitude	99	66	0.494 (0.317–0.772)*	0.782 (0.469–1.305)
	Favorable Attitude	66	89	1	1
Food insecurity	Food secured	131	133	1.569 (0.871–2.825)	1.077 (0.541–2.146)
	Food Insecure	34	22	1	1
Family size	<4	148	139	0.998 (0.485–2.052)	1.131 (0.481–2.656)
	>4	17	16	1	1
Income in birr	<2000	35	14	0.299 (0.152–0.590)*	0.395 (0.184–0.845)^a^
	2000–3,500	44	26	0.442 (0.253–0.773)*	0.541 (0.286–1.023)
	>3,500	86	115	1	1

*p < *0.05*, **p < 0.001 *statistically significance level.*

## Discussion

In this study, the prevalence of inadequate dietary diversity practice during pregnancy was 51.6%, which was in agreement with 57.4% in Southern Ethiopia [35] and 57% in Dire Dewa, Eastern Ethiopia [36]. A study from the United States reported that 46% of pregnant women had inadequate dietary practice [37], this was also consistent with current findings.

Studies from Gondar; Northwest of Ethiopia reported that about 60% of pregnant women had inadequate dietary diversity [38]. Another study from Shashemene, South Western Ethiopia also reported a dietary diversity practice of 74.6% [28]. In these cases, a lower proportion was reported. The observed difference might be due to differences in sample size and time of the investigation across the studies.

Much lower dietary diversity scores were reported by studies 19.6% in Kenya [6]and 19.9% in Gojjam, Northwest Ethiopia [39]. The dietary diversity practice in this study was much higher than in those studies. Such variations might be associated with differences in time of investigation and population.

A study from Malaysia [40] reported a much higher 74% dietary practice than the current study reported. Such variations might be due to differences in population demographics and access to various dietary sources across the population.

In this study, maternal education was statistically associated with dietary diversity, which was supported by the studies from Ghana [41], Kenya [7], Tanzania [42], and Ethiopia [28]. People that are more educated had good dietary diversity compared to those of uneducated in that the more they had an exposure to different nutritional knowledge and information, the higher they tend to use it.

The other factor statistically associated with dietary diversity was maternal knowledge about dietary diversity during pregnancy. Knowledge can affect positively or negatively the practice, through shaping the attitude of people. This was evidently observed in this study that mothers who had poor dietary diversity knowledge had inadequate dietary diversity, which thus was supported by the studies from Malawi [43], and Ethiopia [29].

Additionally, the other factor statistically associated with dietary diversity was monthly income. Those pregnant women who had higher monthly income were more likely to have had adequate dietary diversity compared to their counterparts. This was supported by the studies from Iran [44] and Kenya [7]. This study has the following limitations; The 24 h dietary might not indicate the usual dietary diversity of the pregnant women as the cross sectional nature of the study. The study might have recall and social desirability bias. Moreover, the study used a lower proportion to compute the sample size.

### Conclusion

A higher prevalence of inadequate dietary diversity practice during pregnancy was observed in the study settings. Educational status, knowledge, and monthly income were factors identified to affect the dietary diversity during pregnancy. Being illiterate, primary education, poor dietary knowledge, and lower monthly incomes were associated with inadequate dietary diversity during pregnancy in the study settings.

## Data Availability

A finding of this study was generated from the data collected and analyzed on the basis of stated methods and materials hence all data were already available in the article.

## Abbreviations

AOR, Adjusted Odds Ratio; COR, Crude Odds Ratio; DDS, Dietary Diversity Score; WHO, World Health Organization
